# In utero exposure to polychlorinated biphenyls is associated with decreased fecundability in daughters of Michigan female fisheaters: a cohort study

**DOI:** 10.1186/s12940-016-0175-3

**Published:** 2016-08-31

**Authors:** Lisa Han, Wei-Wen Hsu, David Todem, Janet Osuch, Angela Hungerink, Wilfried Karmaus

**Affiliations:** 1 Department of Pathology, University of Chicago Pritzker School of Medicine, Chicago, IL USA; 2 Department of Statistics, Kansas State University, Manhattan, KS USA; 3 Department of Epidemiology and Biostatistics, College of Human Medicine, Michigan State University, East Lansing, MI USA; 4 Departments of Surgery and Epidemiology and Biostatistics, College of Human Medicine, Michigan State University, 909 Fee Road Room 632, 48824 East Lansing, MI USA; 5 (formerly of) Department of Epidemiology and Biostatistics, College of Human Medicine, Michigan State University, East Lansing, MI USA; 6 Division of Epidemiology, Biostatistics, and Environmental Health, School of Public Health, University of Memphis, Memphis, USA

**Keywords:** PCB, DDE, Fecundability, Offspring, In utero, Fisheaters, Endocrine disruption, Fertility

## Abstract

**Background:**

Multiple studies have suggested a relationship between adult exposures to environmental organochlorines and fecundability. There is a paucity of data, however, regarding fetal exposure to organochlorines via the mother’s blood and fecundability of adult female offspring.

**Methods:**

Data from a two-generation cohort of maternal fisheaters was investigated to assess female offspring fecundability. Serum concentrations of polychlorinated biphenyls (PCBs) and 1,1-bis-(4-chlorophenyl)-2,2-dichloroethene (DDE) in Michigan female anglers were serially measured between 1973 and 1991 and used to estimate *in utero* exposure in their female offspring using two different methods. The angler cohort included 391 women of whom 259 provided offspring information. Of 213 daughters aged 20–50, 151 participated (71 %) and provided information for time intervals of unprotected intercourse (TUI). The daughters reported 308 TUIs (repeated observations), of which 288 ended in pregnancy. We estimated the fecundability ratio (FR) for serum-PCB and serum-DDE adjusting for confounders and accounting for repeated measurements. An FR below one indicates a longer time to pregnancy.

**Results:**

Compared to serum-PCB of <2.5 μg/L, the FR was 0.60 for serum-PCB between 2.5–7.4 μg/L [95 % confidence intervals (CI) 0.36, 0.99], and 0.42 [95 % CI 0.20, 0.88] for serum-PCB >7.4 μg/L. Similar results were obtained using the alternative statistical method to estimate *in utero* serum-PCB. The association was stronger for TUIs when women planned a baby;* FR =* 0.50 for serum-PCB between 2.5–7.4 μg/L, [95 % CI 0.29, 0.89], and 0.30 [95 % CI 0.13, 0.68] for serum-PCB >7.4 μg/L. There was no relationship between *in utero* exposure to DDE and fecundability in daughters.

**Conclusions:**

Decreased fecundability in female offspring of fisheaters was found to be associated with PCB exposure *in utero,* possibly related to endocrine disruption in the oocyte and/or other developing organs influencing reproductive capacity in adulthood.

**Electronic supplementary material:**

The online version of this article (doi:10.1186/s12940-016-0175-3) contains supplementary material, which is available to authorized users.

## Background

The Great Lakes basin and its wildlife are polluted with several halogenated compounds, including polychlorinated biphenyls (PCBs) and 1,1,1-trichloro-2,2, bis (p-chlorophenyl) ethane (DDT) [1, 2]. Anglers in this region have higher serum concentrations of PCBs and the metabolite of DDT, 1,1-bis-(4-chlorophenyl)−2,2-dichloroethene (DDE) than the general U.S. population due to relatively higher fish consumption [2]. These organochlorines (OCs) are lipophilic and bioaccumulate in the food chain, and many have half-lives of a decade or longer [3]. Exposure to these substances occurs by intake of fish, breast milk, and meat, including poultry [3]. Although banned in the U.S. since the 1970s, blood concentration of these substances in humans continue to be measurable, albeit not at the levels previously seen [1].

OCs are classified as endocrine disrupters and have both estrogenic and anti-estrogenic properties. Although the mechanisms of action of these chemicals at varying blood concentrations are only partially known, estrogenic-like OCs are well-known to exhibit their physiological effects at even low doses, similar to other estrogenic steroid hormones [4]. Exposure to OCs may result in adverse health effects, especially in populations with high concentrations of fish consumption and particularly in women with high exposure to OCs who are of child-bearing age and may become pregnant [5–9].

Multiple reproductive effects of adult exposure to PCBs and DDE in women have been studied, including effects on menstrual cycle phase length [10, 11], ability to conceive [12], stillbirth and spontaneous abortion rates [13], fertility as measured by time to pregnancy [14, 15], sex ratio changes in offspring [16], and development of diseases such as endometriosis, uterine fibroids, and polycystic ovarian syndrome [17]. However, there is a dearth of human literature concerning the effect of these chemicals on reproductive health outcomes in exposed offspring. This is especially pertinent because several authors have demonstrated that OC exposure can affect the maturation of the oocyte during the vulnerable phase of folliculogenesis [18–20], which may lead to fertility problems in adult life. This phenomenon occurs through a variety of cellular mechanisms, some unrelated to the well-recognized estrogenic or anti-estrogenic effects of OCs [19, 21, 22].

Three publications investigating fecundability of women exposed to OCs in utero have been published, all using data from the Child Health and Development Studies (CHDS), established in 1959 by the School of Public Health at the University of California at Berkley to investigate health determinants across the lifespan [23]. In 2003, Cohn *et al.* reported on in utero DDT and DDE exposure in 289 eldest daughters of the CDHS cohort and demonstrated that increasing exposure to DDT was associated with a decreased fecundability ratio (FR) (longer time to pregnancy), while increasing exposure to DDE was associated with an increased FR (shorter time to pregnancy) [24]. In a subsequent report by the same research team, studying the same 289 daughters, in utero exposure to individual PCB congeners was associated with both an increased and decreased FR, depending on the congener under investigation [25]. Most recently, the research team of the CDHS grouped PCB congeners according to Wolff’s classification, which is based on known mechanisms of action [26] and reported that the dioxin-like, anti-estrogenic PCB congeners and the group not previously classified was associated with a longer time to pregnancy, and that the non-dioxin anti-estrogenic group and the phenobarbital-inducer groups were associated with a shorter time to pregnancy [27].

The purpose of this study is to investigate the association of in utero exposure to DDE and PCBs on female offspring’s ability to conceive using a unique population of daughters of Michigan fisheaters exposed to lower concentrations of these chemicals than those studied in the CHDS. We hypothesize that even at lower concentrations of exposure seen in our population compared to those reported previously, that in utero PCB and DDE exposure will continue to exert effects on reproductive capacity. If shown, the results would be generalizable to women of reproductive age whose mothers were exposed to PCB and/or DDE at the time of their daughter’s gestation.

## Methods

### Concepts and definitions

**Fig. 1:**
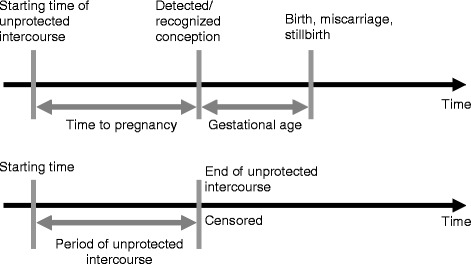
Assessment of time to pregnancy (TTP) and period of unprotected intercourse (PUNP)

**Fig. 2:**
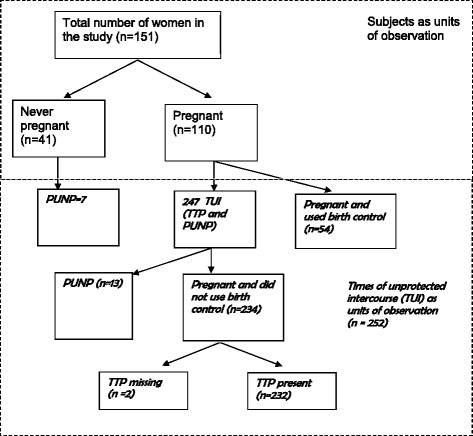
Distribution of Time of Unprotected Intercourse (TUI) in Female Offspring of the Fisheater Family Health Study, 2000–2001. The sample included 151 daughters who provided information on 308 time periods of unprotected intercourse (TUI). The ever-pregnant women had 301 time periods of intercourse resulting in pregnancy, but 54 pregnancies occurred in spite of using birth control (TTP cannot be calculated). Of the 247 TUIs in the ever-pregnant group, 234 ended in pregnancy and 13 did not result in a conception (PUNP). Complete reproductive information was available for 232; however, in two TUIs the time to pregnancy was missing. Of the former, one resulted in a current pregnancy, 168 in a life birth, seven in multiple births, 36 in miscarriages, five in atopic pregnancies and 14 in abortions. One women did not provide information on the pregnancy. The 41 women who were never pregnant contributed seven PUNPs. In total, we had data for 252 TUIs (232 TTPs + 13 PUNPs + 7 PUNPs). TUI: Time of unprotected intercourse, TTP: Time to pregnancy, PUNP: Periods of unprotected intercourse not or not yet leading to pregnancy

Fecundity refers to the biological capacity to give birth to a living child, whereas fecundability is a measure of the probability of conception within one menstrual cycle of unprotected intercourse, whether or not the pregnancy was planned [28]. One measure of fecundability that has been widely used by environmental epidemiologists is time to pregnancy (TTP), measured as the inverse of either the time span or the number of menstrual cycles of unprotected intercourse before conception occurred [29]. To account for periods of unprotected intercourse not or not yet leading to pregnancy, we use the acronym PUNP (Figs. 1 and 2). The time of unprotected intercourse (TUI) describes all periods of unprotected intercourse, whether or not leading to a pregnancy (TTP + PUNP) (Figs. 1 and 2). The fecundability ratio (FR) estimates the odds of attaining pregnancy within a menstrual cycle, given no pregnancy during the previous cycle, for groups of different OC concentrations, compared with the respective reference group.

### Population

Details regarding the study population and methods have been described previously [30]. Briefly, between 1973 and 1991, fisheaters and their spouses were recruited in 11 Michigan shoreline communities at three different time points. During each sampling period and following written informed consent, interviews were conducted using a standardized questionnaire and serum was collected. In the first recruitment period (1973–1974) 156 participants were recruited, in the second period (1979–1982) 1255 participants were enrolled (1,140 new), and in the third period (1989–1991) 717 participants from the previous 1255 were reenrolled (57.1 %) and 11 new participants were recruited. These 728 participants constituted the parental or F0 generation for this study. Serum-PCBs were measured during the first recruitment period, and serum-DDE and serum-PCBs were measured during the second and third periods.

In 2001–2002, following institutional review board approval, we identified 618 mothers from the parental cohort who were of childbearing age between the years 1950 and 1980, and who had both PCB and DDE blood concentrations measured at least once. Of those, 398 (64 %) signed a written consent agreeing to provide information about their daughters aged 20 to 50 years (*n =* 213). We mailed informational brochures and contacted each of the daughters by telephone to explain the study. Of 213 eligible daughters, 151 (71 %) agreed to participate in interviews regarding their reproductive history by written informed consent.

### Questionnaires and classification of time of unprotected intercourse

Participants were interviewed for demographic, medical, gynecologic and reproductive history. Inquiries included past history of genital tract infections, pelvic inflammatory disease, infertility treatment history, and history of a surgical or medical procedure resulting in sterility. Recall aids illustrating the calendar timeline of possible life events and pregnancies were mailed to the participants before the interview. Information on dates (month, year) of the starting time (s) of unprotected intercourse, conception, and delivery was collected, including all pregnancies at the time of the interview, or in cases in which no conception occurred, the total time of unprotected intercourse. For each TUI, we asked the frequency of sexual intercourse, the smoking status of the participant and her partner, and the amount of caffeinated and alcoholic beverages each consumed at the beginning of the TUI. For each pregnancy, we asked whether or not the pregnancy was planned, the gestational age at birth, and whether the couple was using birth control at the time of conception. If pregnancy occurred, we confirmed the data we had collected on TUI by inquiring on the length of time that it took to get pregnant. We used calendar time rather than number of menstrual cycles as it was previously demonstrated that women prefer to recall long TUIs using this approach [31]. We ascertained if there was any interval of 2 months or more during which participants were having intercourse without doing anything to avoid pregnancy and still did not become pregnant. We determined the frequency of sexual intercourse at the beginning of each TUI by prompting participants for frequency by day, week, or month.

Because both TTP and PUNP contribute to a TUI, conception probabilities include both pieces of information. Depending on the responses provided by each study participant, the following four methods were used to calculate TUIs:

1. Information about the duration of TTPs and PUNPs directly provided by the participants;
2. Calculation of TTPs using the month of conception and subtracting it from the starting date of the period of unprotected intercourse;
3. Calculation of TTPs by subtracting the gestational age provided by the participant from the day the pregnancy ended and then calculating the interval back to the couple’s starting time of unprotected intercourse; and
4. Calculation, in cases of PUNPs, of the duration of the TUI by subtracting the starting date from the date the period of unprotected intercourse ended, or if still ongoing at the date of the interview, from the interview date.

An illustration of the use of the interview data to assess the durations of the TTPs and the PUNPs is shown in Fig. 1, and the distribution of each TUI is demonstrated in Fig. 2.

### Determination of serum DDE and PCB in the first generation cohort (parents)

Maternal non-fasting serum PCB and DDE concentrations were determined using the Michigan Department of Community Health modification of the Webb-McCall packed column gas chromatography method described elsewhere [32, 33]. Lipid-unadjusted values were reported. For PCBs, the sum based on the Aroclor 1260 standard were reported, but no individual congeners were determined. Values reported as less than the detectable limit for Aroclor 1260 (3 μg/L) were assigned a concentration of 1.5 μg/L. Less than 5 % of the samples were below the threshold. The technical detection limit for DDE was 1 μg/L. All samples were above this value.

### In utero exposure assessment

Since we did not have measurements from offspring or their mothers at the time of birth, an extrapolation method published by Karmaus et al in 2004 [34] was used to estimate in utero exposures to DDE and PCB. Repeated maternal serum measurements and questionnaire data from each recruitment period was used to construct two linear regression models [34]. Construction of these models used thirty maternal serum OC measurements from the first period of recruitment (1973–1974), 237 from the second period (1979–1982), and 178 from the third period (1989–1991). For the period of 1979–1991, variables in the model estimating PCB exposure included maternal PCB serum concentrations from 1989–1991, the number of years between the serum measurement and the birth of the child, and the number of preceding births (Additional file 1). For the period of 1973–1982, variables in the model estimating PCB exposure included maternal measurements from 1979 to 82, the number of years between the serum measurement and the birth of the child, and the number of years of previous fish consumption (Additional file 1). The two regression lines predicted past PCB serum concentrations with high reliability as measured by intraclass correlation coefficients (ICCs) (ICCs = 0.77 for 1979–1991, and 0.89 for 1973–1982 [35], (Additional file 1), and more accurately estimated actual OC values than two other approaches previously described [36]. Predicted values were higher in the 1979–82 measurements, as indicated by the positive sign for the number of years that passed between 1989–91 and 1979–82. The second formula has a negative sign for the number of years that passed between serum measurements and birth of the child, indicating lower PCB values for 1979–1982 and before. The estimates mirror the trends that were detected for PCB concentrations in fish, which peaked around 1970 but whose peak was delayed approximately 10 years in humans [34]. The same calculations were used for DDE estimates, as only minor differences between the two chemicals were found (data not shown).

We assessed the robustness of our results using the Karmaus method to an alternative method of estimating in utero PCB and DDE exposures and their effects on adult offspring TUIs (subsequently referred to as the mixed models approach). We used a joint model for survival and longitudinal data coupled with a two-stage estimation approach. At the first stage, we formulated a linear mixed effects model to describe the longitudinal profiles of PCBs and DDE, taking into account the number of years of fish consumption and the baseline age at initial measurement time for PCBs and DDE. Subject-specific (as opposed to population average) estimates of these longitudinal models were used to predict the in utero serum concentrations of DDE and PCBs. At the second stage, the predicted values of in utero serum concentrations of DDE and PCBs were subsequently used as explanatory variables in the weighted survival model for TUIs. The weights, computed as the inverse of product of standard errors of the predicted PCBs and DDEs, were invoked to incorporate the variability of these estimates obtained at the first stage into the second stage analysis. Ignoring this variability generated from the first stage analysis is apt to produce biased inferences for the effects of in utero exposure to DDE and PCBs on TUIs. For deliveries that occurred +/−2 years of actual measurements (10 with PCB and 9 with DDE serum concentrations), we compared maternal organochlorine serum-OC at birth and the predictions of their daughters’ OC exposure at birth using the two approaches as shown in Table 2.

### Classification of exposures and confounders

We classified the female offspring exposures using the Karmaus method of analysis based on the estimated DDE and PCB concentrations of their mothers into: 0–2.4 μg/L, 2.5–7.4 μg/L, and 7.5 μg/L and higher. These values reflect those used in multiple publications by the same author in the past and were employed for consistency of result comparisons. The mixed models approach divided estimated PCB and DDE exposures into tertiles of <15.19 μg/L, 15.19–26.87 μg/L, and >26.87 μg/L. Potential confounders at the beginning of the TUI for both methods included a history of pelvic and genital infections; female offspring smoking and offspring partner smoking [37] and female alcohol and caffeine consumption. These were chosen because they are recognized variables that are related to the outcome [15]. Pelvic and genital tract infections were defined as risk factors whenever these disorders occurred before the starting date of a particular TUI. Confounders were classified as yes/no responses for a history of pelvic and genital infections and female offspring and partner smoking. We categorized the consumption of alcohol (12 oz. of beer, 6 oz. of wine, 1 oz. of liquor, mixed drinks and cocktails) into three groups-no alcohol consumption, 0.01–0.5 drinks/day, and >0.5 drinks/day. We assumed that each of these drinks provides an equal amount of alcohol (approximately 12 g) [38]. We categorized caffeine intake (mg per day) into 0–100 mg, 101–300 mg, 301–500 mg based on the participant’s consumption of tea (50 mg/cup), coffee (115 mg/cup), and caffeinated beverages (40 mg/cup) [39].

The participants were grouped by age at the starting time of each TUI into 14–19 years, 20–24 years, 25–29 years, and 30 years and above and by their birth dates into calendar periods as follows: 1950–1954, 1955–1964, and 1965–1980. Information on whether or not the pregnancy was planned at the beginning of TUI was collected as yes or no.

### Statistical analysis

All analyses were performed in SAS 9.4 using the procedure PHREG with options “COVS (aggregate)” and “ties = EXACT”. TUIs without conception were treated as right censored data. As some women experienced multiple TUIs, a Cox proportional hazards model coupled with the sandwich variance estimator was used to account for the within-woman association in TUIs [40]. We computed the fecundability ratio (FR) across the exposure groups (in utero exposure to DDE and PCBs) using the estimated hazard ratio, adjusting for confounders. The exact maximum likelihood estimation was invoked to handle ties. Adjustment by stratification was used because the proportional hazard assumption was violated across the levels of calendar period of birth, age and education. Lastly, we analyzed a model that included only women who planned to conceive. All *P* values reported are two-sided.

## Results

Summary statistics for variables used to estimate serum-OC in the daughters at the time of birth using the Karmaus method are shown in Table 1. The average elapsed time between the last PCB maternal measurement and the birth of the daughter(s) was 16.3 years [95 % CI-2.1 to 30.9 years). Some daughters in the cohort were born after the last maternal measurement (*n =* 15) (data not shown). The comparison of the two approaches using maternal serum-OCs at birth to predict their daughters’ OC exposure concentrations at birth shows substantial correlation, (Spearman rank correlation > 0.8, Table 2).

**Table 1:** Variables used to extrapolate serum-OCs at birth in daughters of the Fisheater Family Health Study

Factors used for exposure assessment	*n =* periods of TUI	Measured in			Median	5 % value	95 % value
		1973–74	1979–82	1989–91			
PCB (1973–74) μg/L	2	x			7.0	7.0	7.0
PCB (1973–74) μg/L	12	x	x		8.0	3.0	22.0
PCB (1979–82) μg/L		x	x		19.2	1.5	34.6
DDE (1979–82) μg/L	12	x	x		22.7	4.4	51.8
PCB (1979–82) μg/L	56		x		9.1	3.0	47.5
DDE (1979–82) μg/L	56		x		11.1	5.1	34.5
PCB (1979–82) μg/L	171		x	x	8.5	3.1	25.4
PCB (1989–91) μg/L			x	x	8.0	4.0	25.7
DDE (1979–82) μg/L	171		x	x	10.3	5.0	29.6
DDE (1989–81) μg/L			x	x	6.9	3.4	21.7
PCB (1973–74) μg/L	11	x	x	x	8.0	4.0	15.0
PCB (1979–82) μg/L		x	x	x	16.6	9.9	25.9
PCB (1989–91) μg/L		x	x	x	21.7	5.6	22.7
DDE (1979–82) μg/L	11	x	x	x	18.4	11.6	30.4
DDE (1989–81) μg/L		x	x	x	10.8	10.3	14.3
					Mean		
Time between measurement and birth (years)	252				16.3	5.5	26.3
Number of siblings	252				1.4	0	4
Years of fish consumption before the birth of the index child	252				3.9	0	23

*OC* organochlorines

*PCB* polychlorinated biphenyls, Aroclor 1260 standard

*DDE* 1,1-bis-(4-chlorophenyl)−2,2-dichloroethene

**Table 2:** Comparison of two approaches to predict daughter birth serum-OC based on maternal serum-OC^a^

Daughter No.	Measurement of maternal serum-OC (μg/L)	The time of daughter’s birth before (−)/after (+) maternal measurement (in years)	Predictions for daughters’ serum-OC at birth (μg/L)	
			Karmaus et al. approach (2004)	Mixed Models approach
PCB				
1	11.0	1.49	6.66	10.92
2	6.4	−1.84	3.31	6.48
3	6.6	−1.52	3.48	5.84
4	16.3	−0.08	9.19	15.42
5	10.2	−0.37	5.70	8.88
6	1.5	−1.07	0.67	1.23
7	4.8	−0.06	2.70	4.90
8	4.5	1.63	2.63	4.66
9	5.0	1.03	2.97	5.63
10	3.0	1.70	1.96	3.34
			r_spearma*n =*_ 0.988 (*p <* 0.001)	
DDE				
2	12.8	−1.84	6.93	13.58
4	19.8	−0.08	11.17	19.87
5	9.1	−0.37	5.08	9.14
6	4.3	−1.07	2.25	4.32
7	4.5	−0.06	2.53	4.54
8	6.2	1.63	2.07	6.24
10	5.7	1.70	0.00	4.59
11	6.9	−1.52	3.65	6.93
			r_spearma*n =*_ 0.810 (*p =* 0.0149)	

^a^Focused on estimations that were +/−2 years before or after actual measurements in the Fisheater Family Health Study, 2000–2001

The characteristics of the daughters are shown in Table 3. Of the 151 study participants, 110 were pregnant at least once. A greater proportion of never-pregnant women were single and younger at the time of the interview than the ever-pregnant women. Of this former group, only 7 of the 41 women had attempted to conceive. In these, the age of first attempt at conception was older than in ever-pregnant women (data not shown).

**Table 3:** Description of Female Offspring of the Fisheater Family Health Study, 2000–2001

Characteristics	Women ever pregnant (*n =* 110) (%)	Women never pregnant (*n =* 41) (%)
Birth cohort		
1950 to 1954	16.4	5.3
1955 to 1965	47.3	19.5
1966 to 1980	36.4	78.1
Age at the time of the interview		
20–25 years	2.8	29.3
25.1–30 year	8.2	24.4
30.1–40 year	41.8	29.3
> 40 year	47.3	17.1
Education		
High school graduate and less	11.0	4.9
Associate degree	31.2	26.8
College graduate or graduate school	57.8	68.3
Marital status		
Married	84.6	39
Single but living together	0.9	12.2
Single	4.6	43.9
Others (engaged, divorced)	10.0	4.9
Mother ate sport-caught fish (information form the offspring)		
Yes	62.7	68.2
No	10.9	22.0
Do not know	26.4	9.8
Breastfed as an infant		
Yes	41.8	43.9
No	50.9	43.9
Don’t know	7.3	12.2
Ever had fertility problems (offspring)	21.8	7.5
In utero DDE exposure (estimated serum concentration in the mother in μg/L)		
0–2.4	33.6	26.8
2.4–7.4	33.6	43.9
> 7.4	31.8	29.3
In utero PCB exposure (estimated serum concentration in the mother in μg/L)		
0–2.4	49.1	39.0
2.4–7.4	28.2	41.5
> 7.4	22.7	19.5
Age at the beginning of the first TUI^a^		
14–< 20 years	17.5	0.0
20–< 25 years	28.2	29.3
25–< 30 years	40.8	24.4
≥ 30 years	13.6	46.3

^a^ *n =* 103 ever-pregnant /41 never-pregnant women

A flow diagram of the distribution of TUIs in the daughters is provided in Fig. 2. The daughters provided information for 308 time periods of intercourse. The median recall duration for the first attempt to conceive was 12.9 years. The geometric mean of the TUIs was 3.1 months (data not shown). The ever-pregnant women had 301 time periods of intercourse resulting in pregnancy, but 54 were using birth control and were therefore not considered in the TUI analysis. Of the remaining 247 TUIs in the ever-pregnant group, 234 ended in pregnancy; complete reproductive information was available for 232. Thirteen TUIs of the ever-pregnant women did not end in pregnancy. The 41 women who were never pregnant contributed seven PUNPs. In total, we had data for 252 TUIs (232 TTPs + 13 PUNPs + 7 PUNPs, Fig. 2). The final analysis was restricted to 249 TUIs because confounding information was missing in 3 participants.

We had a total of 232 TTPs and for each, we compared the duration of the TTP using methods 1–3 described above. Duration to the TTP was directly provided by all women (method 1); we were not able to apply method 2 for two TTPs as the starting time for unprotected intercourse was not recalled, and we did not apply method 3 for another two TTPs which had no defined month to describe when the pregnancy ended prematurely. Of the 230 comparisons that could be made to assess TTP duration by more than one method, there were five estimations that had a discrepancy of more than 1 month. In these cases, we reviewed all reproductive dates and corrected values if two methods resulted in similar durations and the third was inconsistent. We called these women again to clarify the dates and times. In addition, we had a total of 20 PUNPs and used methods 1 and 4 described above for comparisons; all information was available and no disagreement of more than 1 month was found.

The distribution of confounders by OC exposure for each time period of unprotected intercourse (TUIs as compared with individual participants) is shown in Table 4. As expected, the predicted PCB and DDE exposures at the time of birth were highly correlated by both the extrapolation method (r_Spearma*n =*_ 0.59, *p =* 0.001) and the joint model method (r_Spearma*n =*_ 0.59, *P =* 0.001). The median age when the female offspring first attempted to conceive was 26 years. Women with a medium PCB exposure (2.5–7.4 μg/L) were older, 27.3 years, when they first attempted to conceive, which is also seen in the age distribution of the TUIs in Table 4.

**Table 4:** Distribution of Confounders by OC Exposure for Each Time of Unprotected Intercourse in Female Offspring^a^

	Total (*n =* 252)	In utero exposure to PCB (μg/L)				In utero exposure to DDE (μg/L)			
		(0–2.4) (*n =* 144)	(2.5–7.4) (*n =* 66)	(≥7.5) (*n =* 44)	*P* ≤ 0.05 (χ^2^)	(0–2.4) (*n =* 101)	(2.5–7.4) (*n =* 89)	(≥7.5) (*n =* 62)	*P* ≤ 0.05 (χ^2^)
Age at TUI:									
14–20 year	8.3	11.1	4.6	4.8		10.9	10.4	1.6	
20.1–25 years	18.3	18.8	6.1	35.7		13.9	21.8	21.0	
25.1–30 year	38.9	35.4	50.0	33.3		42.6	28.7	46.8	
> 30 year	34.5	34.7	39.4	26.2	^a^	32.7	39.1	30.7	^a^
Cigarette smoking (yes)	30.3	29.9	25.8	38.6		31.7	25.8	35.5	
Offspring’s partner smoking (yes)	32.3	30.1	31.8	39.5		35.6	26.4	35.8	
Caffeine Intake/day									
0–100 mg	46.1	47.9	39.4	50.0		46.5	52.8	33.9	
101–300 mg	27.6	23.6	40.9	20.5		30.7	16.7	38.7	
> 300 mg	26.4	28.5	19.7	29.6		22.8	30.3	27.4	^a^
Alcohol intake (drinks/day)									
no	44.9	42.4	50.0	45.5		44.6	49.4	38.7	
0.01–0.5	36.6	36.1	43.9	27.3		35.6	33.7	41.9	
> 0.5	18.5	21.5	6.1	27.3	^a^	19.8	16.9	19.4	
Pelvic and genital infections before TUI (yes)	62.6	60.4	63.6	68.2		57.4	56.2	79.0	^a^
Offspring’s Education									
High school	9.5	9.0	9.1	11.4		6.9	16.9	3.2	
Associate degree	32.7	37.5	21.2	34.1		34.7	30.3	33.9	
Graduate and more	57.9	53.5	69.7	54.6		58.4	52.8	62.9	
Sexual frequency									
1–14times/month	75.3	75.5	69.4	82.8		72.9	83.6	71.4	
> 14times/month	24.7	24.5	30.6	17.2		27.1	16.4	28.6	
Planning a baby									
yes	79.2	73.2	89.4	83.3		69.3	85.9	87.1	
no	20.8	26.8	10.6	16.7	^a^	30.7	14.1	12.9	^a^

^a^Fisheater Family Health Study, 2000–2001

Although frequency of sexual intercourse was a significant predictor of fecundability, planning of a baby was not. Neither variable disturbed the association between the OC and TUI and were therefore not considered in the final analysis (Table 5). Using the extrapolation method of exposure assessment utilizing repeated measures, controlling for covariates, and taking repeated measurements of TUIs into account, the FR was significantly decreased in both categories of PCB serum concentrations (2.5–7.4 μg/L: *FR =* 0.60, *P =* 0.048; >7.4 μg/L; *FR =* 0.42, *P =* 0.022, Table 5) compared to the reference group.

**Table 5:** Fecundability Ratio for In Utero Exposure to DDE, PCB, and Confounders, Karmaus Method^a^

	All times of unprotected intercourse (*n =* 249)		TUI^b^ ≥ 1 month (*n =* 207)		Women who planned a baby (*n =* 196)	
Variable	FR^e^	95 % CI	FR^e^	95%CI	FR^e^	95%CI
Serum-DDE:^c^						
2.5–7.4 μg/L	1.61	0.90, 2.86	1.36	0.67, 2.76	1.62	0.88, 2.97
> 7.4 μg/L	1.67	0.69, 4.07	1.35	0.42, 4.34	1.72	0.67, 4.43
Serum-PCB:^c^						
2.5–7.4 μg/L	0.60	0.36, 0.99	0.73	0.40, 1.33	0.50	0.28, 0.89
> 7.4 μg/L	0.42	0.20, 0.88	0.58	0.20, 1.67	0.30	0.13, 0.68
Pelvic and genital infections before the TUI	1.00	0.62, 1.63	0.84	0.47, 1.49	1.02	0.59, 1.76
Smoking at the beginning of TUI	0.59	0.21, 1.64	0.43	0.13, 1.42	0.79	0.17, 3.63
Partner smoking at the beginning of TUI	2.09	1.20, 3.63	2.13	1.07, 4.25	1.96	0.90, 4.28
Alcohol consumption at the beginning of TUI (no. of drinks/day) ^d^						
0.01–0.5	0.95	0.57, 1.59	1.01	0.55, 1.87	0.94	0.54, 1.63
> 0.5	1.40	0.64, 3.09	0.94	0.32, 2.78	1.33	0.51, 3.44
Caffeine consumption at the beginning of TUI (mg/day) ^d^						
100–300	0.72	0.43, 1.23	0.78	0.42, 1.44	0.74	0.40, 1.37
> 300	0.91	0.54, 1.53	1.01	0.55, 1.89	0.68	0.34, 1.35

^a^Female Offspring of the Fisheater Family Health Study, 2000–2001

^b^TUI-times of unprotected intercourse, leading or not leading to pregnancy

^c^Reference: <2.5 μg/L

^d^Reference: no

^e^Additionally controlling for age (14–20 years, 25–30 years, and 30 years and above) at the beginning of TUI, the birth cohorts

(1943–1952, 1953–1962, 1963–1972, 1973–1982) and also Education (High school and less, Associate degree, College and above) as stratified variables

As the intention of having a baby was significantly different in the exposure groups (Table 4), we stratified the analysis for planning a baby. In couples in whom the women planned to have a baby, the association between PCB and fecundability was stronger than in the total sample of TUIs (Table 5), but DDE exposure had no significant influence on fecundability in this subgroup.

The estimated model parameters and standard errors from the first step of the joint model analysis using estimated linear mixed effects models are shown in Additional file 2. For DDE, the baseline age and the time in the study were significant, as were the variances of random intercept and random slope, indicating the model captured the time-varying correlations within subject. Similarly, the time in the study and the variances of random intercept and random slope were significant for PCBs, indicating the time-varying correlations within subject were taken account in the model.

The results of the mixed model show similar fecundability estimations to the Karmaus method using repeated measures for estimating in utero exposure to DDE and PCB (Additional file 3). Higher PCB serum concentrations are related to a longer time of unprotected intercourse prior to pregnancy, whereas no effect was seen with DDE exposure.

## Discussion

Our findings, using two different methods to estimate in utero exposure to OCs, demonstrate that in utero exposure to PCBs was significantly associated with decreased fecundability in female offspring of Great Lakes fisheaters. The association was stronger in TUIs when women planned a baby (79.2 % of all TUIs).

The in utero exposure to DDE did not show any effect on fecundability.

Unlike the CHDS, our study included women who had more than one TUI. In order to account for the within-woman association in TUIs, we used a Cox proportional hazards model coupled with the sandwich variance estimator. The inclusion of all TUIs contributed to the reliability of the findings. An additional strength of this study is that the original estimates of exposure using the Karmaus method were verified using a mixed modelling approach, which also showed that higher PCB serum concentrations were related to reduced fecundability ratios (longer times of unprotected intercourse before a pregnancy occurred). Both estimates of exposure yielded results comparable to those reported from the CHDS cohort [24, 25]. The differences in estimation of OC exposure in the daughters using either the Karmaus or mixed model method as demonstrated in Table 2 can be partially explained by the fact that the two models use very different statistical frameworks. In addition, the Karmaus method took PCB half-lives into account, whereas this was not possible using the mixed model method. Interestingly, the latter method most closely estimated actual maternal OC values (Table 2). Ranking the predicted OC serum concentrations using the two methods, however, gave very similar results (PCB and DDE r_spearma*n =*_ 0.988, *p <* 0.0001 and 0.810, *p =* 0.0149, respectively.

Our study has some important limitations to consider. Our small sample size (*n =* 151) restricts us from detecting weak alterations in fecundability. Another limitation is that we had no information on the age, BMI, or OC exposure of the partner of our participants, all of which could affect couple fecundity [14]. In contrast to the CHDS which had a direct measurement of in utero exposure in relation to the child’s birth (1–3 days after the birth of offspring) [24], our study estimated the in utero exposure using maternal DDE and PCB measurements from three different time points, and most, but not all of the participants were born before the OC measurements were taken. In addition, our maternal OC serum concentrations were not adjusted for lipids.

We reported previously that estimated maternal OC concentrations using the Karmaus method significantly contributed to the OC burden present in their adult daughters measured, on average, 40 years after exposure. The proportion of variance for PCBs and DDE explained by maternal serum concentrations was 50 % and 18 %, respectively [30]. Fecundability of the daughters may have therefore been partially affected by OC exposures encountered after birth, and two recent studies have reported that reduced fecundability is associated with increasing PCB serum concentration in adulthood [14, 15].

Prior research has demonstrated that since OCs are lipophilic, they can be transferred to offspring by breastfeeding [3]. Our questionnaire queried mothers to ascertain whether offspring were breastfed, and included duration of breastfeeding when the answer was affirmative. In a previous publication, we demonstrated that exposure to increasing DDE serum concentrations decreases both initiation and duration of breastfeeding [41]. Since our cohort was exposed to both PCB and DDE, we did not include breastfeeding as a confounding variable, as we consider it a possible intermediary, appearing to be in the path of DDE ➔ breastfeeding ➔ offspring exposure.

In their earlier study, Cohn et al found that in utero DDT and DDE exposure was associated with decreased and increased fecundability, respectively [24]. We were unable to investigate DDT exposure because maternal serum concentrations in our cohort were quite low, but our results for DDE trend towards their findings. As expected, serum concentrations of lipid-unadjusted DDE were lower in the Fisheaters’ cohort compared to the CHDS cohort due to different measurement time points: median values of DDE in our cohort were approximately 8.5 μg/L [30] compared to 48.19 μg/L [24] in the CHDS cohort. For lipid-unadjusted PCB serum concentrations between the two cohorts, the trends were similar: median total PCB values were approximately 7.7 μg/L [30] in the Fisheaters’ cohort and approximately 37.6 μg/L in the CHDS cohort [25]. Nonetheless, our overall results replicated one another, suggesting that there are fecundability consequences to in utero PCB exposure even at low serum concentrations.

In the second report examining in utero OC exposure and fecundability in the CHDS, the investigators found that some PCB congeners increased fecundability of the daughters, (105, 138 and 183) while others decreased it (187, 156 and 99), and that infertility rates were higher in daughters exposed to PCB congeners associated with longer TTPs [25]. Although PCB congeners were not measured in maternal serum samples in our study (owing to technical limitations secondary to use of an Aroclor standard), we have previously reported serum concentrations of PCB congeners in their daughters as measured during adulthood [42]. The serum of our adult daughters contains more PCB congeners than those reported in the CHDS, and in different frequencies, making it very difficult to compare our two studies in this regard, especially because it is well-known that the PCB congeners are correlated with one another. To account for this in the CHDS, Gennings et al [27] incorporated a nonlinear weighted quartile sum approach, which can be very robust in evaluating extreme values of chemical concentrations and therefore provide reliable estimates of exposure for the model. We were unable to incorporate this approach because in utero exposure of PCB congeners was not measured. In addition, in the CHDS, of those congeners found to be significantly associated with fecundability (187, 156, 99, 105, 138, and 183), the frequency above the limits of detection was 57–100 %, depending on the congener. In contrast, these same congeners were above the limits of detection in our adult daughters at much lower frequencies (27.3, 40.3, 7.2, 5.8. 54.7, and 5.8 %, respectively), making inferences between the two studies even more difficult.

Retrospective cohort studies are particularly subject to selection bias, depending on the number of subjects who are eligible compared to the number that participate. Of the 213 eligible female offspring of the Michigan Fisheaters Cohort, 151 (71 %) participated in this study. To explore the possibility of selection bias, we compared the PCB serum concentrations measured in those mothers whose daughters participated or did not participate in this follow-up study. There was no significant difference (data not shown), making selection bias unlikely.

Reproductive epidemiology studies are plagued with specific biases well-described by Weinberg et al [43]. The behavior modification bias refers to the fact that women who have difficulty conceiving may seek to decrease smoking, caffeine or alcohol intake in response to fertility problems. For each TUI, we therefore incorporated these three important covariates into the models, which reduces, but does not eliminate, this kind of bias. We also analyzed the participants in the subgroup who planned to have a baby (*n =* 196, Table 3). Women who planned their pregnancies had FRs that were similar to those for all TUIs (Table 3).

A time trend bias results in spurious associations when exposure and time to pregnancy follow a comparable secular trend. We evaluated whether changes in intra-uterine PCB exposure between 1950 and 1980 coincided with a trend in the number of TUIs that offspring born in 5 year intervals between these dates experienced. The median extrapolated maternal PCB serum concentrations (and the respective median TUIs) were: 1950–54: 5.3 μg/L (3 months), 1955–59: 1.8 μg/L (4.5 months), 1960–64: 1.6 μg/L (2 months), 1965–69: 1.6 μg/L (3 months), 1970–74: 2.1 μg/L (2 months), 1975–80: 1.3 μg/L (3 months); data not shown. Since the median TUI varies by the six time periods studied without any trend depending on exposure concentrations, a time trend bias is unlikely.

A planning bias, present when less fertile couples are more careless about using birth control, may result if carelessness is distributed differently in exposure groups. To investigate a potential planning bias, we calculated the proportion of birth control failures (54/308) for the incremental categories of PCB exposure, and did not detect a significant difference in distribution of birth control failures by PCB serum concentrations (data not shown). This finding suggests that our results are not affected by planning bias.

Weinberg et al considers the conception rate within the first cycle following discontinuation of contraception to represent an unbiased estimate of fecundability [43]. The wantedness bias addresses the definition of contraceptive failure by participants, or put another way, intentionality of pregnancy. In order to investigate, we conducted a sub-analysis, excluding TUIs of ≤ 1 month. Women who had birth control failures in the sub-analysis (*n =* 42, TUI > 1 month) had fecundability ratios (FRs) that were similar to those for all TUIs (data not shown). Therefore, a wantedness bias is unlikely.

A pregnancy recognition bias results when in some exposed groups spontaneous abortions are treated as normal menstrual periods thus resulting in longer TUIs. We observed 36 of 231 TTPs (15.6 %) which resulted in spontaneous abortions. The rates of recognized spontaneous abortions in the three exposure groups were not statistically significantly different (data not shown), reducing the possibility of bias of a differential recognition.

A medical intervention bias enhances the probability of conception. To guard against this bias, we additionally censored all TUIs > 12 months, the time at which the couple is defined medically as infertile. Based on this analysis, we found the following fecundability ratios: for PCB (2.5–7.4 μg/L: *FR =* 0.493, *P =* 0.007; >7.4 μg/L: *FR =* 0.30, *P =* 0.008; data not shown). Hence, it is unlikely that medical intervention biased the finding of reduced fecundability related to prenatal PCB exposure.

## Conclusions

To the best of our knowledge, this is the second cohort study which reports on the association between intra-uterine organochlorine exposure and fecundability in offspring. Our results support the hypothesis of low in utero endocrine disrupter exposure having adverse effects on fecundability in adulthood, possibly related to endocrine disruption in the oocyte or in other endocrine organs directly associated with reproduction, including the uterus, fallopian tubes, thyroid, hypothalamus, and pituitary glands [25]. Additional research on the influence of gestational organochlorine exposures on the fecundity of offspring is warranted. It is important to consider multiple exposures to toxicants, investigate different PCB congeners, and disentangle the different strengths of their associations.

## Additional files

Additional file 1: Karmaus Extrapolation of Maternal Serum-PCB at Delivery in the Fisheater Family Health Study, 2000–2001. (DOC 27 kb) Additional file 2: Estimated model parameters and standard errors for the first stage linear mixed models. (DOC 39 kb) Additional file 3: Fecundability Ratio for In Utero Exposure to DDE, PCB, and Confounders, Mixed Models Method. (DOC 35 kb) Additional file 4: Dataset supporting conclusions of fecundability manuscript. (CSV 34 kb)

## Abbreviations

CHDS: Child health and development studies
CI: Confidence intervals
DDE 1: 1-bis-(4-chlorophenyl)-2, 2-dichloroethene
DDT 1: 1, 1-trichloro-2, 2, bis (p-chlorophenyl) ethane
FR: Fecundability ratio
ICCs: Intraclass correlation coefficients
OCs: Organochlorines
PCBs: Polychlorinated biphenyls
PUNP: Periods of unprotected intercourse not or not yet leading to pregnancy
TTP: Time to pregnancy
TUI: Time intervals of unprotected intercourse
