# Supplementary material for: In utero exposure to polychlorinated biphenyls is associated with decreased fecundability in daughters of Michigan female fisheaters: a cohort study
Source: Environ Health. 2016 Aug 31;15(1):92. doi: 10.1186/s12940-016-0175-3 (PMC5006410; doi:10.1186/s12940-016-0175-3)
Supplement: Additional file 1: — Karmaus Extrapolation of Maternal Serum-PCB at Delivery in the Fisheater Family Health Study, 2000–2001. (DOC 27 kb) [file 12940_2016_175_MOESM1_ESM.doc]

**Additional file 1:** Karmaus Extrapolation of Maternal Serum-PCB at Delivery in the

Fisheater Family Health Study, 2000-2001

| Extrapolation period | Formula derived from linear regression analyses | Intraclass correlation coefficient |
| --- | --- | --- |
| 1979 to 1991 | PCB = 10*(-0.193)  + log10 (PCB 1989/91 survey) * 0.781  + years between measurement & birth * 0.049  - number of preceding births * 0.145 | 0.77  lower 5% limit: 0.71 |
| 1973 to 1982 | PCB = PCB in the 1979/82 survey * 0.565  - years between measurement & birth * 0.163  + years of preceding fish consumption * 0.106 | 0.89  lower 5% limit: 0.80 |
