# Supplementary material for: In utero exposure to polychlorinated biphenyls is associated with decreased fecundability in daughters of Michigan female fisheaters: a cohort study
Source: Environ Health. 2016 Aug 31;15(1):92. doi: 10.1186/s12940-016-0175-3 (PMC5006410; doi:10.1186/s12940-016-0175-3)
Supplement: Additional file 2: — Estimated model parameters and standard errors for the first stage linear mixed models. (DOC 39 kb) [file 12940_2016_175_MOESM2_ESM.doc]

Additional file 2: Estimated model parameters and standard errors for the first stage linear

mixed models #

|  | **DDE** | | |  | **PCB** | | |
| --- | --- | --- | --- | --- | --- | --- | --- |
| **Parameter** | **Estimate** | | **Standard Error** |  | **Estimate** | | **Standard Error** |
| **Regression Coefficients** |  |  |  |  |  | |  |
| Intercept | 7.71 | * | 3.529 |  | -10.714 |  | 5.500 |
| Age at baseline (years) | 0.239 | ** | 0.054 |  | 0.316 | ** | 0.059 |
| Time in the study (years) | -0.879 | ** | 0.147 |  | -0.033 |  | 0.058 |
| Fish consumption (0-21 years) | 0.338 |  | 3.208 |  | 8.869 |  | 4.951 |
| Fish consumption (21-70 years) | 0 | ¥ | - |  | 8.058 |  | 4.851 |
| Fish consumption (70+ years) | - |  | - |  | 0 | ¥ | - |
| **Variance-Covariance parameters** |  | |  |  |  | |  |
| Variance (RI) | 650.960 | ** | 61.441 |  | 108.980 | ** | 11.063 |
| Covariance (RI, RS[Time]) | -51.250 | ** | 5.087 |  | -2.280 | ** | 0.735 |
| Variance (RS[Time]) | 4.455 | ** | 0.477 |  | 0.348 | ** | 0.085 |
| Variance (Measurement Error) | 2.703 |  | 5.954 |  | 7.985 | ** | 2.436 |

# From the Fisheater Family Health Study, 2000-2001

* p-value<0.05; **: p-value<0.01; ^¥^: reference serum concentration.

Note: RI is random intercept and RS[Time] is random slope with respect to time.
