# Supplementary material for: In utero exposure to polychlorinated biphenyls is associated with decreased fecundability in daughters of Michigan female fisheaters: a cohort study
Source: Environ Health. 2016 Aug 31;15(1):92. doi: 10.1186/s12940-016-0175-3 (PMC5006410; doi:10.1186/s12940-016-0175-3)
Supplement: Additional file 3: — Fecundability Ratio for In Utero Exposure to DDE, PCB, and Confounders, Mixed Models Method. (DOC 35 kb) [file 12940_2016_175_MOESM3_ESM.doc]

Additional file 3: Fecundability Ratio for *In Utero* Exposure to DDE, PCB, and Confounders, Mixed

Models Method#

|  | **All times of unprotected intercourse**  **(n=249)** | |
| --- | --- | --- |
| **Variable** | **FR^†^** | **95% CI** |
| Serum-DDE:^Φ1^ |  |  |
| 15.19 – 26.87 μg/L | 0.51 | 0.23, 1.11 |
| > 26.87 μg/L | 1.85 | 0.84, 4.10 |
| Serum PCB:^Φ2^ |  |  |
| 5.35 - 10.30 μg/L | 0.41 | 0.15, 1.10 |
| > 10.30 μg/L | 0.39 | 0.16, 0.91 |
| Pelvic and genital infections before the TUI | 0.61 | 0.35, 1.07 |
| Smoking at the beginning of TUI | 2.18 | 0.26, 18.6 |
| Partner smoking at the beginning of TUI | 1.25 | 0.43, 3.66 |
| Alcohol consumption at the beginning of TUI (no. of drinks/day)^ψ^ | | |
| 0.01 - 0.5 | 0.78 | 0.34, 1.79 |
| > 0.5 | 1.26 | 0.53, 3.00 |
| Caffeine consumption at the beginning of TUI (mg/day)^ψ^ | | |
| 100 - 300 | 1.37 | 0.81, 2.32 |
| >300 | 1.06 | 0.49, 2.27 |

^#^ From the Fisheater Family Health Study, 2000-2001

^¥^ TUI – times of unprotected intercourse, leading or not leading to pregnancy

^Φ1^ 33.3th and 66.6th percentiles of predicted DDE serum concentration were 15.19 and 26.87 μg/L. Reference: <15.19 μg/L

^Φ2^ 33.3th and 66.6th percentiles of predicted PCB serum concentrations were 5.35 and 10.30 μg/L. Reference: <5.35 μg/L

^Ψ^ Reference: no

† Additionally controlling for age (14-20 years, 25-30 years, and 30 years and above) at the beginning of TUI, the birth cohorts (1943-1952, 1953-1962, 1963-1972, 1973-1982) and also Education (High school and less, Associate degree, College and above) as stratified variables.
